# 

**Published:** 1903-02

**Authors:** 


Fifty-Eighth Year.

VOL. LVIII. Whole Number. DCLXXV.

FEBRUARY, 1903.

New Series. VOL. XLII No. 7

### BUFFALO MEDICAL JOURNAL

Established 1845 by AUSTIN FLINT, M. D.

EDITOR:

WILLIAM WARREN POTTER, M. D.

ASSISTANT EDITORS:

WILLIAM C. KRAUSS, M. D. NELSON W. WILSON, M. D.

ASSOCIATE EDITORS:

JAMES WRIGHT PUTNAM, M. D. ERNEST WENDE, M. D.
JOHN PARMENTER, M. D. JOHN A. MILLER, Ph. D
HARVEY R. GAYLORD, M. D. MAUD J. FRYE, M. D.

LIBRARY
SURGEON GENERAL'S OFFICE
[illegible] FEB 1903

SCRIPTA FERUNT ANNOS

EUROPEAN AGENCY, J. B. BAILLIERE & FILS, 19 Rue Hautefeuille, Paris.
*Subscription, if paid in advance, $2.00; otherwise, $2.50. Foreign subscription, $2.50*
Entered at the Post Office at Buffalo, N. Y., as second-class mail matter.

A. T. BROWN PRINTING HOUSE, BUFFALO, N. Y.

Table of Contents on Page V.

